# Supplementary material for: Metagenomic investigation of bacterial laccases in a straw-amended soil
Source: PeerJ. 2025 Apr 28;13:e19327. doi: 10.7717/peerj.19327 (PMC12045287; doi:10.7717/peerj.19327)
Supplement: Supplemental Information 1 [file peerj-13-19327-s001.docx]

**Table S1.** Summary for metagenomic assembly and annotation

|  | Number | Size |
| --- | --- | --- |
| **Assembly feature** |  |  |
| Scaftigs | 416,112 | 306 Mb |
| Scaftigs N50 |  | 692 bp |
| Scaftigs N90 |  | 525 bp |
| Longest scaftig |  | 22.6 kb |
| Scaftigs average |  | 735.41 bp |
| **Metagenome anotation** |  |  |
| ORFs | 329,007 | 199 Mb |
| Integrity:end | 72,133 (21.9%) |  |
| Integrity:none | 100,109 (30.4%) |  |
| Integrity:all | 51,935 (15.8%) |  |
| Integrity:start | 104,830 (31.9%) |  |
| ORFs average |  | 605.4 bp |
| ORFs GC content |  | 63.48% |

**Table S2**. Alignment of bacterial laccases against NCBI-NR database

| Laccase | Cloning Method | Closest relative | Acession number | Identity |
| --- | --- | --- | --- | --- |
| S_14142 | mTAIL-PCR | multicopper oxidase family protein [Betaproteobacteria bacterium] | HEU60661 | 62.79% |
| S_23069 | mTAIL-PCR | multicopper oxidase family protein [Phycisphaerales bacterium] | HEG44379 | 65.46% |
| S_63994 | directly cloned | copper oxidase [Betaproteobacteria bacterium] | OGA72159 | 70.79% |
| S_230270 | mTAIL-PCR | multicopper oxidase family protein [*Kribbella pittospori*] | TCC54276 | 65.18% |
| S_326900 | mTAIL-PCR | hypothetical protein A3J29_16445 [Acidobacteria bacterium] | OFW39622 | 71.07% |
| S_394411 | mTAIL-PCR | multicopper oxidase family protein [Actinobacteria bacterium] | TML27845 | 64.12% |
| S_507105 | directly cloned | copper resistance system multicopper oxidase [*Vulcaniibacterium* sp. R-5-52-3] | WP_147652669 | 75.64% |

**Table S3.** Sequence identity between different bacterial laccases

|  | S_14142 | S_23069 | S_63994 | S_230270 | S_326900 | S_394411 | S_507105 |
| --- | --- | --- | --- | --- | --- | --- | --- |
| S_14142 | 100% | 23.27% | 51.69% | 35.46% | 27.67% | 34.31% | 27.67% |
| S_23069 |  | 100% | 31.28% | 24.34% | 29.56% | 24.74% | 24.21% |
| S_63994 |  |  | 100% | 35.21% | 29.24% | 36.36% | 27.57% |
| S_230270 |  |  |  | 100% | 28.11% | 70.02% | 25.71% |
| S_326900 |  |  |  |  | 100% | 25.82% | 37.1% |
| S_394411 |  |  |  |  |  | 100% | 25.85% |
| S_507105 |  |  |  |  |  |  | 100% |

**
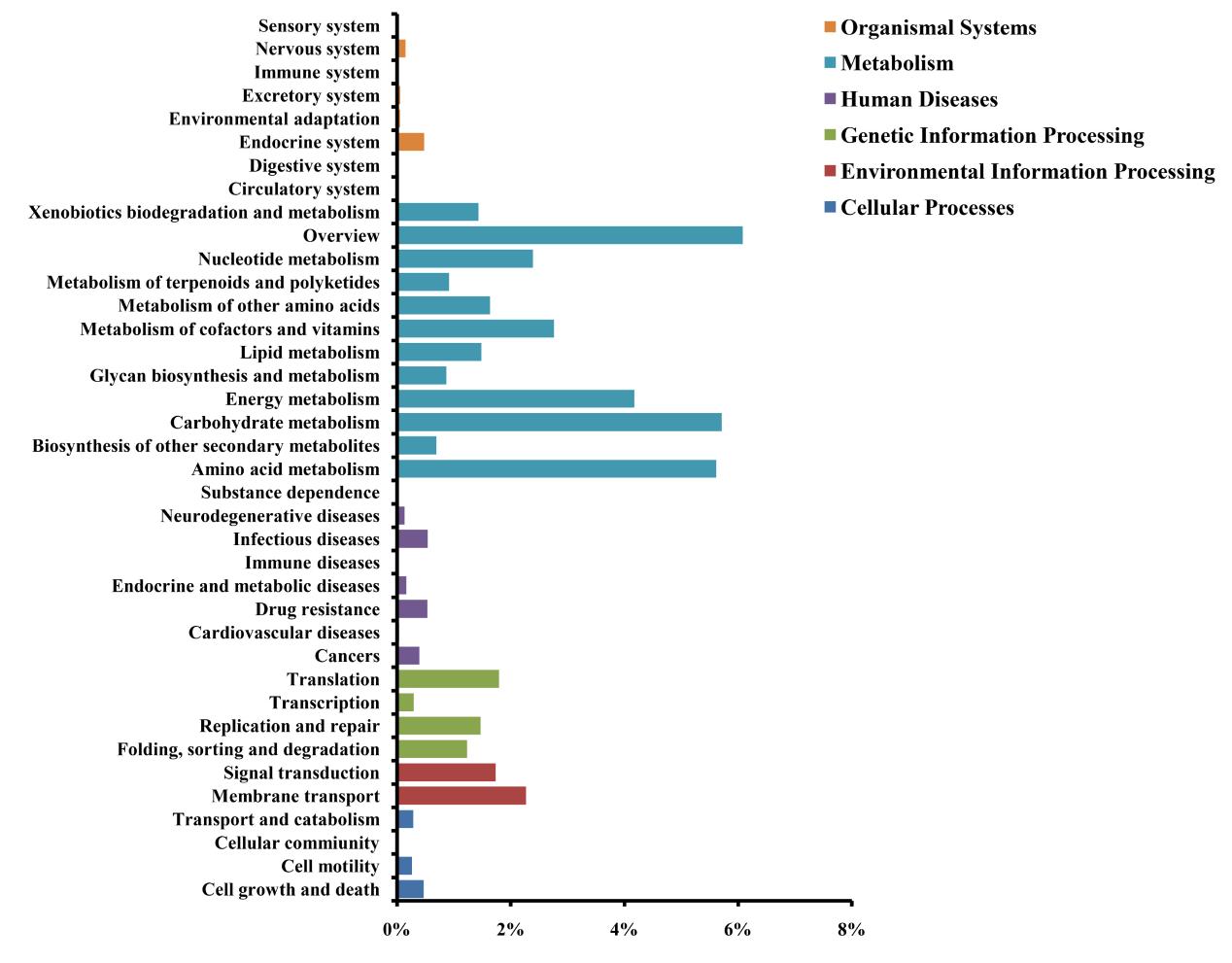
**

**Figure S1.** KEGG functional categories of the straw-amended metagenome.


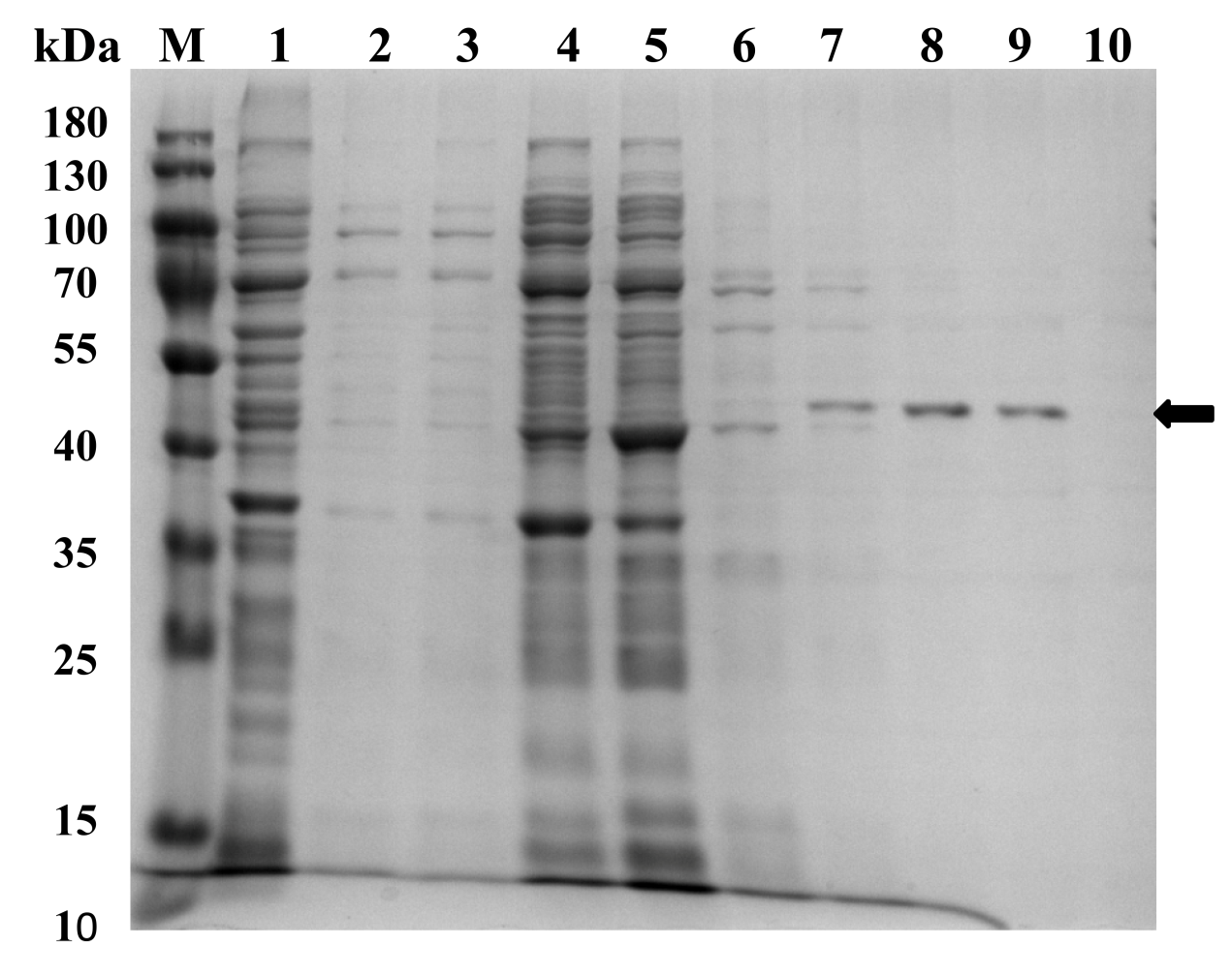


**Figure S2**. Purification of the recombinant lacS1. M. Protein marker; 1. Flow through; 2-10. Elutes by NTA0, NTA20, NTA40, NTA60, NTA80, NTA100, NTA200,

NTA300 and NTA500. Arrow indicates recombinant lacS1.


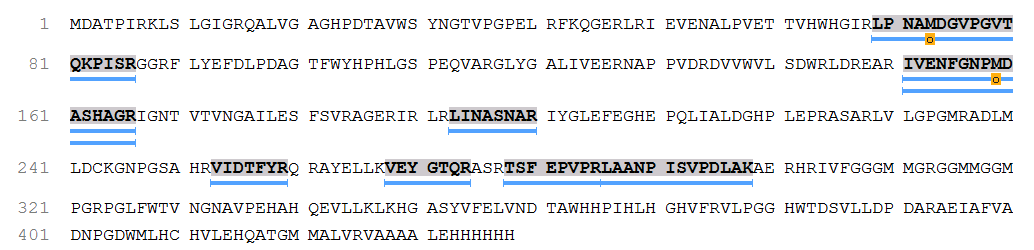


**Figure S3.** LC-ESI-MS/MS analysis of the purified recombinant lacS1
